# Supplementary figures and images for: Improved Swarm Intelligent Blind Source Separation Based on Signal Cross-Correlation
Source: Sensors (Basel). 2021 Dec 24;22(1):118. doi: 10.3390/s22010118 (PMC8747210; doi:10.3390/s22010118)

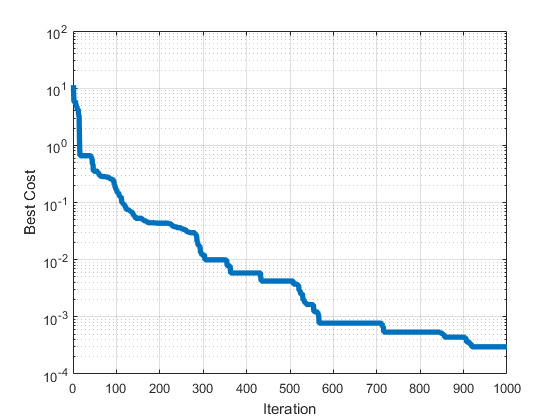

Supplement: Supplementary file 1 [file sensors-22-00118-s001.zip › code/SpeechSeparation/qpso/semilog.png]

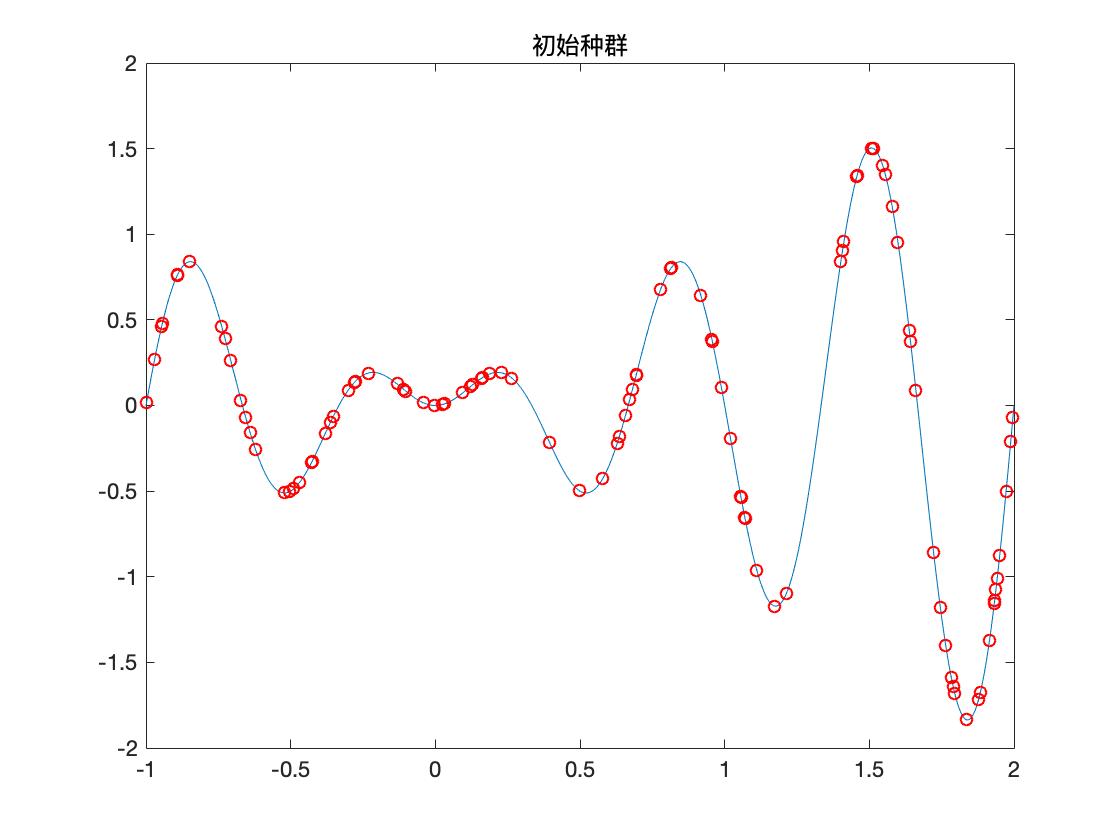

Supplement: Supplementary file 1 [file sensors-22-00118-s001.zip › code/SpeechSeparationW4/GA/初始种群.jpg]

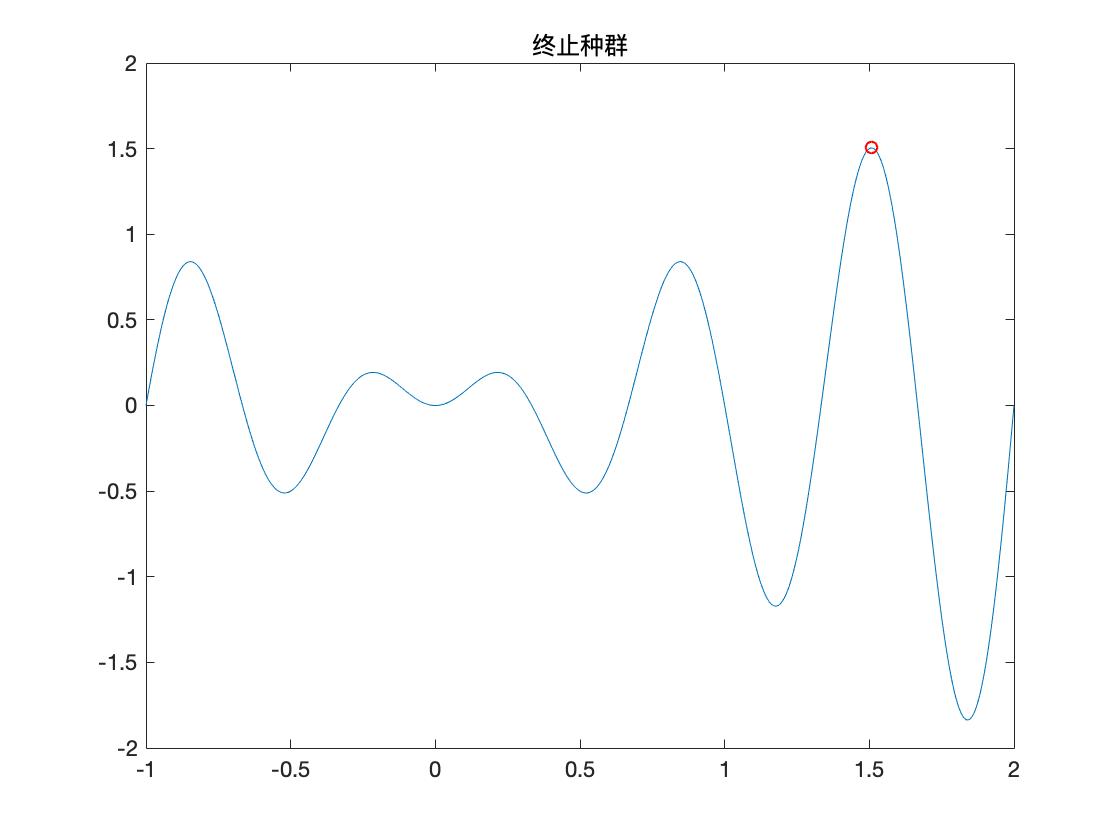

Supplement: Supplementary file 1 [file sensors-22-00118-s001.zip › code/SpeechSeparationW4/GA/终止种群.jpg]
